# Kinesthetic deficits after perinatal stroke: robotic measurement in hemiparetic children

**DOI:** 10.1186/s12984-017-0221-6

**Published:** 2017-02-15

**Authors:** Andrea M. Kuczynski, Jennifer A. Semrau, Adam Kirton, Sean P. Dukelow

**Affiliations:** 1 University of Calgary, Calgary, AB Canada; 2 Section of Neurology, Department of Pediatrics, Alberta Children’s Hospital Research Institute, Calgary, AB Canada; 3 Department of Clinical Neurosciences, Hotchkiss Brain Institute, Calgary, AB Canada; 4 Department of Clinical Neurosciences, Hotchkiss Brain Institute, Foothills Medical Centre, University of Calgary, 1403 – 29th St. NW, Calgary, AB T2N 2T9 Canada

**Keywords:** Perinatal, Stroke, Kinesthesia, Proprioception, Robotics, Cerebral palsy

## Abstract

**Background:**

While sensory dysfunction is common in children with hemiparetic cerebral palsy (CP) secondary to perinatal stroke, it is an understudied contributor to disability with limited objective measurement tools. Robotic technology offers the potential to objectively measure complex sensorimotor function but has been understudied in perinatal stroke. The present study aimed to quantify kinesthetic deficits in hemiparetic children with perinatal stroke and determine their association with clinical function.

**Methods:**

Case–control study. Participants were 6–19 years of age. Stroke participants had MRI confirmed unilateral perinatal arterial ischemic stroke or periventricular venous infarction, and symptomatic hemiparetic cerebral palsy. Participants completed a robotic assessment of upper extremity kinesthesia using a robotic exoskeleton (KINARM). Four kinesthetic parameters (response latency, initial direction error, peak speed ratio, and path length ratio) and their variabilities were measured with and without vision. Robotic outcomes were compared across stroke groups and controls and to clinical measures of sensorimotor function.

**Results:**

Forty-three stroke participants (23 arterial, 20 venous, median age 12 years, 42% female) were compared to 106 healthy controls. Stroke cases displayed significantly impaired kinesthesia that remained when vision was restored. Kinesthesia was more impaired in arterial versus venous lesions and correlated with clinical measures.

**Conclusions:**

Robotic assessment of kinesthesia is feasible in children with perinatal stroke. Kinesthetic impairment is common and associated with stroke type. Failure to correct with vision suggests sensory network dysfunction.

## Background

Proprioception has classically been defined as a combination of both sense of position and sense of motion (kinesthesia) without the use of vision [1–3]. Intact proprioception is essential to provide accurate feedback for motor decisions. Proprioceptive dysfunction has been associated with impairments in the execution of coordinated [4] and reaching movements [5], as well as negatively impacting safety [6] in adult stroke survivors. Kinesthetic function includes aspects in the sense of motion such as the speed, timing, and direction of movement. In adults with stroke, kinesthetic deficits are common, occurring in approximately 60% of individuals [7–9]. However, our understanding of the characteristics and prevalence of kinesthetic deficits following perinatal stroke are far less developed.

Perinatal stroke is a focal vascular brain injury occurring between the 20^th^ week of gestation and the first 28 post-natal days [10]. While perinatal stroke is common, the pathophysiology remains unknown. There are two common types of ischemic perinatal stroke: large arterial ischemic strokes (AIS) which typically occur at term and damage cortical and subcortical structures, and periventricular venous infarctions (PVI), earlier fetal lesions restricted to the subcortical white matter [11]. Both stroke types usually damage major components of the sensorimotor system but location and timing are different. For this reason, sensorimotor impairments for AIS cases are often greater than in PVI [11, 12]. How these two types of perinatal stroke affect proprioceptive function in hemiparetic children is unknown.

Sensory impairments in CP are common in >50% of hemiparetic children, with reported deficits in passive motion sense [13, 14] and visuomotor performance [15]. We recently characterized position sense deficits in children with perinatal stroke, and found correlations with clinical function and greater impairments with AIS relative to PVI [16]. Despite a number of studies of sensory function [14, 17–19], the prevalence and severity of kinesthetic deficits is not well known in children with hemiparetic CP. This is due in part to limited availability of measurement tools for kinesthetic impairments [20–22]. To assess position sense, clinicians commonly move a joint such as the distal-interphalangeal joint in a given direction while the patient closes their eyes. The patient is then asked to report the direction of movement. This technique has been shown to have significant problems with reliability [23] and may only detect more severe impairments. Further, it fails to detect impairments in sensing the speed or amplitude of the movement.

Recent advances in robotic technology offer more objective, accurate and reliable methods for measuring upper limb sensory function than most clinical scales [7, 24–29] and have been widely applied in adult stroke [30, 31]. With slight modification, these techniques have been successfully applied to hemiparetic children [16]. In this study, we quantified kinesthetic function in children with hemiparetic CP secondary to perinatal ischemic stroke using a robotic exoskeleton. We hypothesized that children with perinatal stroke would show impaired kinesthesia compared to healthy controls and that deficits would be greatest in those with AIS compared to PVI. We further hypothesized that the degree of deficit would correlate with standardized clinical function measures.

## Methods

### Participants

Participants with perinatal stroke were recruited from a population-based research cohort (Alberta Perinatal Stroke Project). Children of term birth (≥36 weeks) between 6 to 19 years of age were included. Each participant had clinical confirmation of symptomatic hemiparetic CP (Pediatric Stroke Outcome Measure (PSOM) [32] ≥0.5 and Manual Abilities Classification System (MACS) [33] grade I-IV and child/parent perceived functional limitations) and MRI-confirmed unilateral perinatal stroke according to validated criteria [11]. Participants were excluded if they had severe hemiparesis (MACS grade V), severe spasticity (Modified Ashworth Scale [34] = 4), neurological conditions other than stroke, botulinum toxin injections or upper limb surgery within six months, or were unable to comply with the study protocol. Controls comparable in age and sex with no neurological or orthopaedic impairments were recruited from the community and underwent the same evaluations. All participants provided written consent, parental consent, or assent as appropriate. Methods were approved by the institutional research ethics board.

### Robotic assessment of kinesthetic function

Robotic assessments were performed at Foothills Medical Centre (Calgary). A KINARM robotic exoskeleton (BKIN Technologies Ltd., Kingston, Ontario) assessed kinesthesia as previously described in healthy adults and stroke survivors [7, 8]. Participants were fitted to a modified wheelchair base with each arm supported by the robotic exoskeleton. Modifications were made for smaller children by adding one inch risers to the arm troughs and a booster seat with four inch foam padding to achieve comparable upper limb positioning [16].

**Fig. 1:**
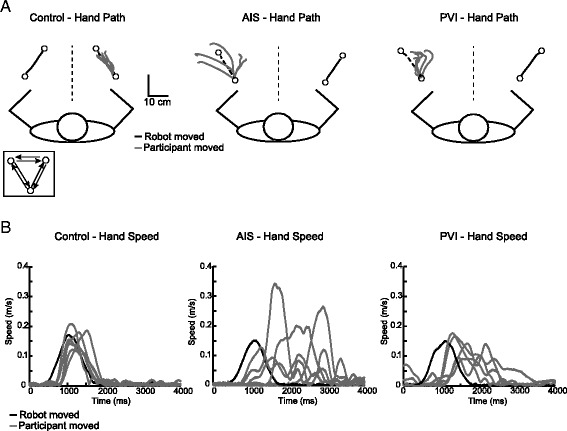
Pediatric robotic kinesthesia task. White circles represent the location of robotic movement endpoints. Each of the three targets were separated by 12 cm. Black lines show the movement of the robotically moved passive arm. Grey lines show the movements performed by the active arm to mirror-match the movement of the robot. **a** Hand paths of an exemplar 10 year old female control, AIS, and PVI participants for six movements in a single direction. A 17 year old male participant with AIS makes larger initial direction errors (IDE) in comparison to the ideal (*robotic*) trajectory (*dashed line*). A 7 year old female participant with PVI also demonstrates greater angular deviations than the exemplar control. **b** Hand speed profiles associated with movement in one direction depict the speed of the robotically moved passive arm (*black line*) relative to movements of the participant (*grey lines*). The speed profile of the exemplar control indicates excellent matching of robot speed. The speed profile of the AIS participant indicates variable speed after the movement of the passive arm. A participant with PVI matches the speed of the robot, but moves much later when matching

The kinesthesia task measured sense of limb motion. Before the start of each trial, the robot moved one of the participant’s arms (passive arm) to one of 3 spatial locations separated by 12 cm with a speed of 0.18 m/s (Fig. 1a). Participants were required to place a virtual white circle representing the index finger of their active arm in a red circle that appeared in the workspace at one of the 3 possible spatial locations, bringing the two limbs to a mirrored start position and initiating the beginning of the trial [7]. In this way, the two upper limbs always began equidistant from the next spatial target. The visual targets were extinguished and as soon as participants felt the robot move their passive arm, they were required to move their opposite, active arm to mirror-match speed, amplitude and direction of movement. Participants were given 10 s to respond; failure to respond was recorded as non-movement. For stroke cases, the robot moved the stroke-affected arm and they mirror matched with their opposite arm. For controls, the robot moved the participant’s dominant arm and they matched with their non-dominant arm. Participants completed 6 blocks of 6 trials (one in each of 6 possible directions) which were ordered pseudo-randomly within a block. Each participant first completed the task with vision of the upper extremities occluded using an apron and shutter, and then completed a second trial with vision restored.

Eight parameters were quantified as described previously [7]. Positional data of the active arm was mirrored across the x-axis for all analyses. For each participant, mean behaviour was taken across all 36 trials to calculate single values for each of the following parameters:

1. *Response latency (RL)*: the difference between time of movement onset for the active and passive arms (in milliseconds). Movement onset was defined as 10% of peak hand speed and positive acceleration of the active arm.
2. *Initial direction error (IDE)*: angular difference (in degrees) between the active and passive arms at peak hand speed.
3. *Peak speed ratio (PSR)*: the ratio of how accurately the subject matched the peak speed of the robotic movement (passive arm). Ratios <1 indicated slower movement of the active arm, >1 indicated faster movement than the passive arm.
4. *Path length ratio (PLR)*: the ratio of how well the active arm matches the length of the robotic movement. Movement end was defined as a reduction in speed to 10% of peak speed of the active hand. Ratios <1 indicated the subject matched with a shorter movement length (>1 indicated longer length).

Variability of each parameter was also calculated as the standard deviation across all movements: RLv, IDEv, PSRv, and PLRv.

For stroke participants, data was compared to normative ranges (95% range of normal data) derived from healthy controls for each parameter. A stroke participant was considered significantly impaired on a given parameter if their score fell outside this control range. In order to determine if a subject was impaired on the task we looked across the eight parameters that were recorded. Five percent of control subjects fell outside the 95% range of normal parameter performance on three or more parameters in the no vision condition, and on two or more parameters in the vision condition. Thus, we operationally defined stroke participants as failing the task if they fell outside the normative range on three or more parameters on the task with vision occluded and two or more parameters on the task with the use of vision.

### Clinical assessment

A series of common clinical sensory tests were performed by the same experienced therapist in a standardized fashion at the beginning of each session.

A. *Upper limb position sense*. The therapist moved the participant’s wrist up and down three times with vision occluded and asked them to identify the direction of the end position. The same assessment was repeated with the thumb. Outcomes were dichotomized as unable to correctly identify position in either thumb or wrist (0) or able (1) to identify.
B. *Thumb localization task (TLT)*. With vision occluded, the therapist moved and positioned the participant’s non-dominant upper limb lateral to the midline and asked the participant to pinch the thumb with their opposite thumb and index finger [35]. The task was scored on a four point scale from 0 (no difficulty locating) to 3 (unable to locate). Outcomes were dichotomized as normal (0) or impaired (>0).
C. *Stereognosis*. Standardized objects (nickel, key, and paperclip) were sequentially placed in the palm bilaterally, starting with the non-dominant hand. With vision occluded, participants were asked to verbally identify the object and scored either 0 (unable to identify), 0.5 (identified category but not object), or 1 (able to identify). Scores were dichotomized as normal (1) or impaired (0) when participants were unable to correctly identify 2 or more objects.
D. *Graphesthesia*. With vision occluded, the assessor used the back of a pen to “draw” a 3, 5, and 7 in the palm bilaterally, starting with the non-dominant hand. The participant was asked to verbally identify the number and scored either 0 (unable to identify) or 1 (able to identify). Scores were dichotomized as normal (1) or impaired (0) if unable to correctly identify 2 or more numbers.

Participants also underwent the following standardized, validated assessments of sensorimotor and visual function:

A. *Assisting Hand Assessment (AHA)* assessed bimanual upper extremity function in children with hemiparetic CP with scores expressed as logit units [36].
B. *Chedoke-McMaster Stroke Assessment (CMSA)* for upper extremity motor recovery was scaled from 1 (flaccid paralysis) to 7 (normal movement) [37].
C. *Purdue pegboard (PPB)* measured hand dexterity bilaterally (LaFayette Instrument Co, LaFayette, IN, USA). Participants picked up one peg at a time and successively filled holes as quickly as possible in 30 s. This test was repeated twice with each limb and the best score was used [38].
D. *Modified Edinburgh Handedness Inventory* determined relative upper extremity dominance using a 10-item questionnaire [39].
E. *Vision.* Visual acuity was assessed using a Snellen eye chart (20/30 minimum required). Visual fields were assessed using confrontation and scored as normal, hemianopsia, or quadrantanopsia.
F. *Behavioural Inattention Test (BIT)* screened participants for visuospatial neglect using 6 conventional subtests with a total possible score of 146 and scores <130 indicative of neglect [40].

### Statistical analysis

Kolmogorov-Smirnov tests determined the normality of data distributions. A one-way ANCOVA with Bonferroni post-hoc correction was used to determine differences between groups on kinesthetic parameters controlling for age. In the control group, the performance on the kinesthesia parameters were fit with linear (IDE, IDEv, PSR, PSRv, PLR, and PLRv) and first order polynomial (RL and RLv) functions, according to R squared values, to address the effects of aging using SigmaPlot (Systate Softeare Inc., San Jose, CA, USA) software. The 95% prediction bands were computed from the mean curve for each parameter. Mann–Whitney U-tests or paired t-tests compared performance between vision conditions within each group. Mann–Whitney U-tests or independent sample t-tests compared kinesthetic performance between left and right hemispheric damage in stroke participants to determine the effects of hemispheric damage. Mann–Whitney U-tests or independent sample t-tests compared kinesthetic performance (no vision) between participants that passed versus failed clinical sensory measures. Pearson’s or Spearman’s correlations with Bonferroni correction assessed the relationship of kinesthetic parameters with clinical measures. Chi-square tests evaluated the association between clinical sensory and robotic measures. Statistical analyses were performed using SigmaPlot (Systat Software Inc., San Jose, CA, USA), SPSS (IBM, Armonk, NY, USA) and Matlab (Mathworks, Natick, MA, USA).

## Results

**Fig. 2:**
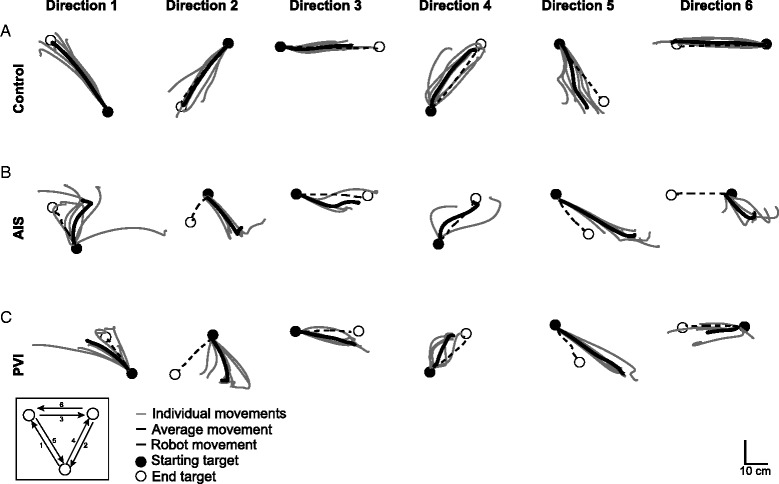
Hand movements in the kinesthesia task. Individual and average hand movements in each direction for an exemplar participant from each group. White circles represent the location of robotic movement endpoints. Black dashed lines show the mirrored movement of the robotically moved passive arm from the first target (*black circle*) to the end target (*white circle*). The direction of movement between the three targets is shown in the bottom left corner. Light grey lines show the movements performed by the active arm to mirror-match the movement of the robot. Dark grey lines indicate the average hand movement in each direction. **a** A 15 year old female typically developing child/adolescent demonstrates excellent matching of the robot movement with low IDE and excellent PLR. **b** An 11 year old female participant with AIS shows difficulty in matching the length (PLR) and direction (IDE) of movement, and does not complete all 6 trials within the movements (direction 3 and 4). **c** A 15 year old male with PVI moves with large IDE in most directions

Demographic and clinical measures of the 149 participants (*n* = 23 AIS, *n* = 20 PVI, *n* = 106 controls) are described in Table 1. All three groups were comparable with respect to age and sex. Figure 1 depicts the kinesthesia task and provides an example of typical performance within each group. Thirteen (57%) AIS and 7 (35%) PVI cases failed the kinesthesia task without vision, while 11 (48%) AIS and 8 (40%) failed the kinesthesia task with vision. All six individual movements as well as an average trajectory for each of the six directions for a typically developing control, arterial and venous stroke participant is shown in Fig. 2. An AIS (Fig. 2b) and PVI (Fig. 2c) participant show more variability in their movements in all six directions, moving with greater IDE in most cases.

**Table 1:** Group characteristics

	AIS (*N* = 23)	PVI (*N* = 20)	Controls (*N* = 106)
Age (years)	12.7 ± 3.7	11.6 ± 3.7	12.3 ± 3.9
Sex	10 F, 13 M	8 F, 12 M	51 F, 55 M
Affected Arm	14 R, 9 L	10 R, 10 L	--
Handedness	11 R, 12 L	11 R, 9 L	95R, 11 L
TLT [0,1,2,3]	[12, 10, 0, 1]	[15, 5, 0, 0]	[105, 1, 0, 0]
Position Sense [0,1]			
Thumb	[11, 11]^a^	[2, 18]	[106, 0]
Wrist	[8, 14]^a^	[1, 19]	[106, 0]
Stereognosis [0,0.5,1]			
Nickel	[16, 3, 4]	[3, 11, 6]	[3, 32, 71]
Key	[18, 0, 5]	[3, 3, 13]^a^	[6, 1, 99]
Paperclip	[18, 0, 5]	[6, 0, 14]	[3, 1, 102]
Graphesthesia [0,1]			
7	[15, 8]	[4, 16]	[10, 96]
5	[14, 9]	[5, 15]	[2, 104]
3	[17, 6]	[9, 11]	[10, 96]
CMSA [1,2,3,4,5,6,7]			
Non-dominant Arm	[0, 0, 12, 2, 3, 3, 3]	[0, 0, 3, 1, 3, 5, 8]	--
Dominant Arm	[0, 0, 0, 0, 0, 4, 19]	[0, 0, 0, 0, 0, 2, 18]	--
Logit AHA [0–100]	61.3 ± 19.9 (32–100)^b^	75.2 ± 16.0 (55–100)^c^	--
MA [0–100]	69.1 ± 21.3 (31–100)^b^	89.4 ± 10.7 (75–100)^c^	--
PSOM motor [0,0.5,1,1.5,2]	[0, 1, 6, 0, 16]	[0, 4, 10, 0, 6]	--
MACS [1–5]	[4, 16, 0, 0, 0]^b^	[8, 5, 0, 0, 0]^c^	--
PPB			
Non-dominant Arm	1.48 ± 3.1 (0–11)	5.65 ± 3.8 (0–11)	13.5 ± 2.2 (8–19)
Dominant Arm	12.6 ± 2.0 (10–16)	13.3 ± 1.8 (10–16)	14.7 ± 2.3 (8–21)
BIT [0–146]	128 ± 24.0 (56–145)^a^	139 ± 4.4 (130–146)^a^	--

Participant age is indicated as a mean ± standard deviation. Results from Thumb Localization Test (TLT), Stereognosis, Graphesthesia, Position Sense, and the Chedoke-McMaster Stroke Assessment (CMSA), Pediatric Stroke Outcome Measure (PSOM), and Manual Abilities Classification System (MACS) are shown as the number of subjects who obtained a given score (square brackets). Assisting Hand Assessment (AHA), Melbourne Assessment (MA), Purdue Pegboard (PPB), and Behavioural Inattention Test (BIT) scores are shown as a mean ± standard deviation, with a range of scores shown in brackets. Abbreviations: *AIS* arterial ischemic stroke, *PVI* periventricular venous infarction, *F* female, *M* male, *L* left, *R* right.^a^ Data missing from one participant;^b^ data missing from 3 AIS participants;^c^ data missing from 7 PVI participants

### Response latency

**Fig. 3:**
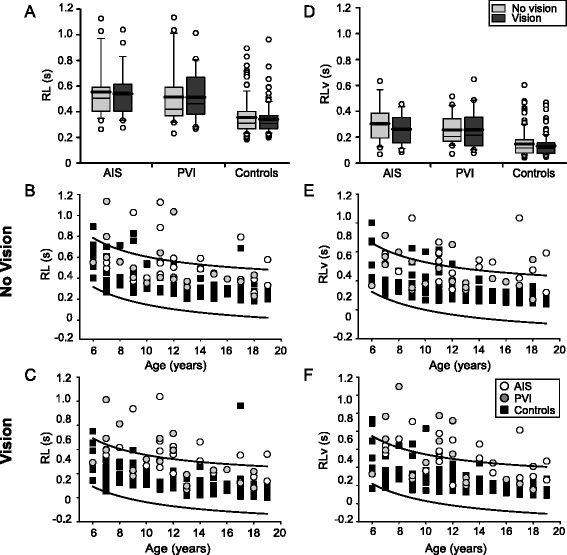
Group data of response latency. Boxplots of response latency (RL) and RLv (*top row*) are shown for each of the three groups with vision removed and vision restored. Scatter plots without (*middle row*) and with (*bottom row*) vision show the performance in the parameters for stroke cases and controls with 95% prediction intervals of control performance defining normal boundaries (*black lines*). Both AIS and PVI groups demonstrate increased RL (**a**) and RLv (**d**) relative to controls. Stroke cases often demonstrated consistently greater RL (**b**, **c**) and RLv (**e**, **f**) across all ages (x-axis)

Stroke participants demonstrated greater response latencies relative to controls (F(2145) = 22.8, *p* < 0.001) (Fig. 3a). Both the AIS (0.55 ± 0.2 s, *p* < 0.001) and PVI (0.51 ± 0.2 s, *p* < 0.001) groups took longer to respond than controls (0.36 ± 0.1 s). AIS and PVI groups were not different from each other. Seven (30%) AIS and 4 (20%) PVI participants fell outside the normal control range for RL (Fig. 3b). Restoration of vision did not affect RL in any group and was still greater in stroke compared to controls (F(2145) = 35.4, *p* < 0.001). Both AIS (0.54 ± 0.2 s, *p* < 0.001) and PVI (0.51 ± 0.2 s, *p* < 0.001) participants showed slower RL compared to controls (0.34 ± 0.1 s) but were not different from each other. Ten (43%) AIS and 6 (30%) PVI participants fell outside the normal range of RL with vision (Fig. 3c).

RLv was also greater in cases than controls (F(2145) = 28.4, *p* < 0.001) (Fig. 3d). AIS (0.30 ± 0.2 s, *p* < 0.001) and PVI (0.26 ± 0.1 s, *p* < 0.001) participants had more variability in RL relative to controls (0.15 ± 0.1 s). RLv did not differ between AIS and PVI without vision. Eleven (48%) AIS and 4 (20%) PVI participants fell outside the normal range of RLv without vision (Fig. 3e). Vision restoration did not change RLv in any group and it remained greater in stroke participants compared to controls (F(2145) = 33.7, *p* < 0.001). AIS (0.26 ± 0.1 s, *p* < 0.001) and PVI (0.26 ± 0.2 s, *p* < 0.001) cases displayed larger RLv than controls (0.13 ± 0.08 s) but did not differ from each other. Ten (43%) AIS and 6 (30%) PVI participants fell outside the normal range of RLv with vision (Fig. 3f).

### Initial direction error

**Fig. 4:**
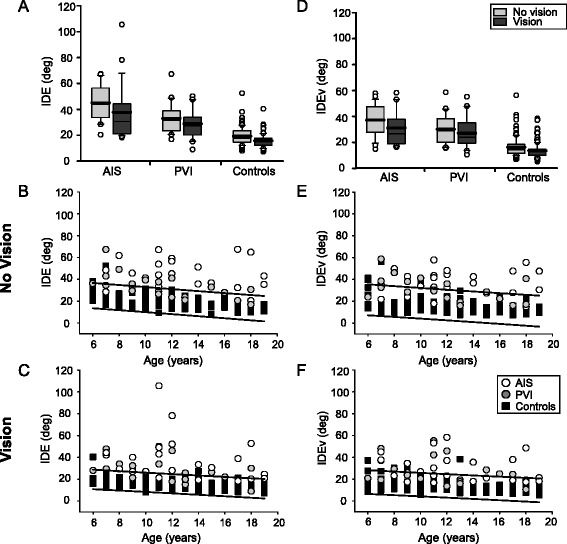
Group data of initial direction error. Boxplots of initial direction error (IDE) and IDEv (*top row*) are shown for each of the three groups with vision removed and vision restored. Scatter plots without (*middle row*) and with (*bottom row*) vision show the performance in the parameters for stroke cases and controls with 95% prediction intervals of control performance defining normal boundaries (*black lines*). Both AIS and PVI demonstrate increased IDE (**a**) and IDEv (**d**) relative to controls in both vision conditions. Stroke cases often demonstrated greater IDE (**b**, **c**) and IDEv (**e**, **f**) across all ages (x-axis)

Stroke cases displayed significantly larger IDE than controls (F(2145) = 108.3, *p* < 0.001) (Fig. 4a). Without vision, the AIS (44.9 ± 13°) group showed larger IDE than PVI (33.0 ± 12°, *p* < 0.001) and controls (19.3 ± 6.8°, *p* < 0.001). PVI participants also demonstrated greater IDE than controls (*p* < 0.001). Nineteen (83%) AIS and 9 (45%) PVI participants fell outside the normal range of IDE with vision occluded (Fig. 4b). Overall, performance improved with restoration of vision. Stroke cases still had greater IDE than controls (F(2145) = 60.4, *p* < 0.001) and AIS (37.7 ± 21°) was greater than PVI (28.9 ± 11°, *p* < 0.01). The PVI group also had greater IDE than controls (*p* < 0.001). Fourteen (61%) AIS and 10 (50%) PVI participants fell outside the normal range of IDE with vision restored (Fig. 4c). The restoration of vision improved IDE in the AIS (*U* = 162, *p* < 0.05) and control (*U* = 3751, *p* < 0.001) groups but not PVI.

With vision occluded, IDEv was greater in stroke compared to controls (F(2145) = 70.4, *p* < 0.001). AIS participants (37.2 ± 12°) displayed greater IDEv than PVI (30.2 ± 11°, *p* < 0.01) and both were greater than controls (16.3 ± 7.7°, *p* < 0.001) (Fig. 4d). Seventeen (74%) AIS and 7 (35%) PVI participants fell outside the normal range of IDEv (Fig. 4e). With vision restored, participants with stroke still had larger IDEv than controls (F(2145) = 59.5, *p* < 0.001). Both AIS (31.1 ± 13°, *p* < 0.001) and PVI (27.1 ± 12°, *p* < 0.001) demonstrated greater IDEv than controls (13.7 ± 5.9°) but were not different from each other. Eleven (48%) AIS and 8 (40%) PVI participants fell outside the normal range of IDEv (Fig. 4f). Restoration of vision improved IDEv in the control group only (*U* = 4237, *p* < 0.01).

### Peak speed ratio

**Fig. 5:**
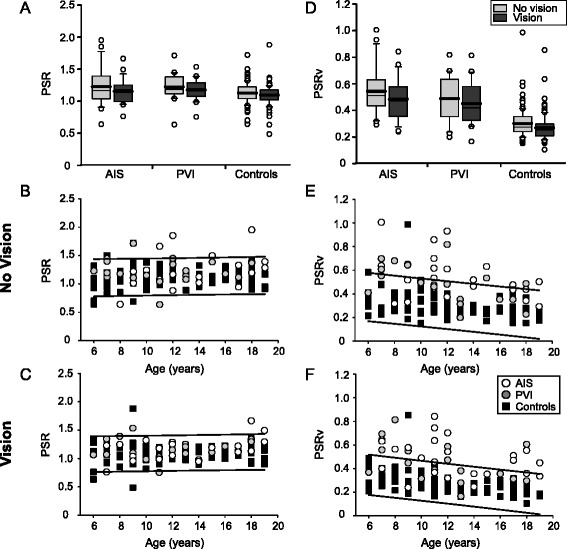
Group data of peak speed ratio. Boxplots of peak speed ratio (PSR) and PSRv (*top row*) are shown for each of the three groups with vision removed and vision restored. Scatter plots without (*middle row*) and with (*bottom row*) vision show the performance in the parameters for stroke cases and controls with 95% prediction intervals of control performance defining normal boundaries (*black lines*). Both AIS and PVI groups demonstrate increased PSR (**a**) and PSRv (**d**) relative to controls. Stroke cases often demonstrated greater PSR (**b**, **c**) and PSRv (**e**, **f**) across all ages (x-axis)

No significant differences were observed between the groups in PSR in either vision condition (Fig. 5a). Six (26%) AIS and 2 (10%) PVI participants fell outside the normal range of PSR in the no vision condition, whereas 2 (9%) AIS and 2 (10%) PVI fell outside the range in the vision condition (Fig. 5b, c). Restoration of vision did not affect PSR. With vision occluded, PSRv was higher in stroke cases compared to controls (F(2145) = 50.0, *p* < 0.001) (Fig. 5d). Both AIS (0.54 ± 0.2, *p* < 0.001) and PVI (0.49 ± 0.2, *p* < 0.001) groups displayed greater PSRv than controls (0.30 ± 0.1) without vision. Thirteen (57%) AIS and 7 (35%) PVI participants fell outside the normal range of PSRv without vision (Fig. 5e). With vision restored, PSRv was greater in cases compared to controls (F(2145) = 54.2, *p* < 0.001). AIS (0.49 ± 0.2, *p* < 0.001) and PVI (0.45 ± 0.2, *p* < 0.001) participants again showed greater PSRv than controls (0.27 ± 0.1). Thirteen (57%) AIS and 9 (45%) PVI fell outside the normal range in the vision condition (Fig. 5f). Restoration of vision improved PSRv in the control group only (*U* = 2091, *p* < 0.01).

### Path length ratio

**Fig. 6:**
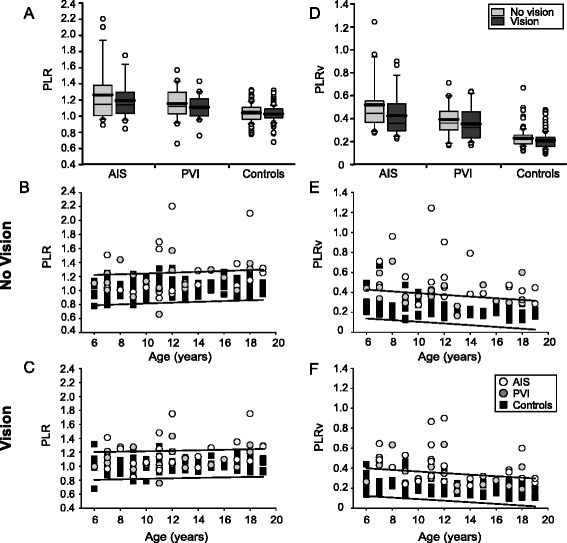
Group data of path length ratio. Boxplots of path length ratio (PLR) and PLRv (*top row*) are shown for each of the three groups with vision removed and vision restored. Scatter plots without (*middle row*) and with (*bottom row*) vision show the performance in the parameters for stroke cases and controls with 95% prediction intervals of control performance defining normal boundaries (*black lines*). Both AIS and PVI groups demonstrate increased PLR (**a**) and PLRv (**d**) relative to controls. Stroke cases often demonstrated greater PLR (**b**, **c**) and PLRv (**e**, **f**) across all ages (x-axis)

Stroke cases demonstrated greater PLR than controls (F(2145) = 15.8, *p* < 0.001). With vision removed, both AIS (1.26 ± 0.35, *p* < 0.001) and PVI (1.16 ± 0.20, *p* < 0.05) participants had greater PLR compared to controls (1.04 ± 0.11) (Fig. 6a). Eight (35%) AIS and 7 (35%) PVI participants fell outside the normal range of PLR in the no vision condition (Fig. 6b). When vision was restored, stroke cases still had greater PLR than controls (F(2145) = 15.3, *p* < 0.001). Both AIS (1.19 ± 0.24, *p* < 0.001) and PVI (1.11 ± 0.15, *p* < 0.05) had greater PLR than controls (1.03 ± 0.10). Nine (39%) AIS and 6 (30%) PVI participants fell outside the normal range of PLR with vision (Fig. 6c). AIS and PVI groups did not differ from each other for PLR in either vision condition.

PLRv was greater in participants with stroke than controls (F(2145) = 63.3, *p* < 0.001) (Fig. 6d). Without vision, AIS (0.52 ± 0.3) demonstrated larger PLRv than PVI (0.39 ± 0.1, *p* = 0.001) and control (0.23 ± 0.08, *p* < 0.001) groups. PVI participants also displayed greater PLRv than controls (*p* < 0.001). Fifteen (65%) AIS and 9 (45%) PVI participants fell outside the normal range of PLRv in the no vision condition (Fig. 6e). With vision restored, PLRv was larger in stroke cases compared to controls (F(2145) = 49.6, *p* < 0.001). AIS (0.43 ± 0.2, *p* < 0.001) and PVI (0.35 ± 0.1, *p* < 0.001) still showed greater PLRv relative to controls (0.21 ± 0.08). AIS participants had greater PLRv than PVI (*p* < 0.05). Twelve (52%) AIS and 8 (40%) PVI participants fell outside the normal range of PLRv (Fig. 6f). Controls, but not stroke particpants, showed improvement in PLRv when vision was restored (*U* = 4662, *p* < 0.05).

### The effect of ipsilesional motor deficits

Given the potential impact of motor deficits in the ipsilesional or “unaffected arm” we specifically examined the six stroke cases (4 AIS, 2 PVI) who were found to have motor impairments in their ipsilesional arm (CMSA score = 6; Table 1). Three AIS cases with ipsilesional motor deficits failed the kinesthesia task without vision, and all four failed the task when vision was restored. Neither PVI participant with ipsilesional motor deficits failed the kinesthesia task without vision. With vision, one PVI case failed the kinesthesia task. When the 6 stroke cases with ipsilesional motor deficits were removed from data analysis, no differences between the stroke and control groups were found in terms of the kinesthetic parameters with or without vision.

With the removal of these 6 stroke cases from the data analysis, group differences between the AIS and PVI groups changed in 2 parameters in both the no vision and vision conditions. Without vision, IDEv differed less between the stroke groups when the ipsilesional cases were removed (*p* = 0.05 rather than *p* < 0.01), and PLRv no longer differed (*p* = 0.5). With vision, IDE did not differ between AIS and PVI groups when participants with ipsilesional deficits were excluded (*p* = 0.06). PLRv was also no longer significant between AIS and PVI groups (*p* = 1.0 versus *p* < 0.05) with the exclusion of participants with ipsilesional deficits.

### Hemispheric lateralization

In the AIS group, comparing participants with left versus right hemispheric damage did not reveal any differences in performance on any of the eight parameters with or without vision. Similarly in the PVI group, comparing participants with left versus right hemispheric damage did not reveal any differences in performance on any of the eight parameters with or without vision.

### Clinical position sense

All controls showed no impairment in clinical sensory assessments with the exception of one 6 year-old who failed the thumb localization task (Table 1). Impaired thumb localization was associated with stroke type where 11 (48%) AIS and 5 (25%) PVI failed compared to controls (0.9%, *p* < 0.001). Kinesthetic robotic task performance without vision did not differ between AIS participants that passed versus failed the TLT.

Impaired clinical position sense (thumb and wrist) was associated with AIS where 13 (57%) failed compared to controls (0%, X^2^(1) = 66.6, *p* < 0.001). AIS participants with impaired position sense displayed greater RLv (0.34 ± 0.3 vs 0.18 ± 0.1, *p* < 0.05) and greater PSRv than participants with normal position sense (0.53 ± 0.5 vs 0.43 ± 0.3, *p* = 0.01). Position sense was impaired in 2 (10%) PVI participants which was more than controls (0%, X^2^(1) = 10.8, *p* = 0.001), but less than AIS (X^2^(1) = 10.2, *p* = 0.001). Kinesthetic parameters (no vision) did not differ between PVI participants that passed versus failed clinical position sense.

### Clinical cortical sensation

Stereognosis was impaired in 18 (78%) AIS participants compared to 2 (2%) controls (X^2^(1) = 84.2, *p* < 0.001). AIS participants that failed stereognosis had greater RL (0.55 ± 0.5 vs 0.39 ± 0.4, *p* < 0.05) and PSRv (0.53 ± 0.5 vs 0.34 ± 0.3, *p* < 0.001). Impaired stereognosis was also associated with PVI where 4 (20%) failed compared to 2 (2%) controls (X^2^(1) = 12.2, *p* < 0.001). PSRv was again greater in PVI subjects that failed stereognosis (0.65 ± 0.1 versus 0.45 ± 0.2, *p* < 0.05).

Graphesthesia was impaired in 16 (70%) AIS participants compared to 5 (5%) controls (X^2^(1) = 58.3, *p* < 0.001). AIS participants with impaired graphesthesia had greater PSRv (0.60 ± 0.2 vs 0.43 ± 0.09, *p* = 0.05). Graphesthesia impairment was also associated with PVI where 5 (25%) failed compared to 5 (5%) controls (X^2^(1) = 9.5, *p* < 0.01). PVI participants with impaired graphesthesia had greater RL (0.60 ± 0.5 vs 0.39 ± 0.4 *p* < 0.05) and IDEv (36.2 ± 35° vs 27.8 ± 23°, *p* < 0.05).

### Clinical outcomes

Logit AHA scores were lower in AIS (61.3 ± 20) compared to PVI (75.2 ± 16) participants (t(31) = −2.04, *p* = 0.05) but were not correlated with kinesthetic parameters. CMSA scores were lower in AIS than PVI participants for the affected limb (*U* = 118, *p* < 0.01). CMSA scores were not correlated with kinesthetic performance. Fine motor dexterity as determined by the PPB was significantly lower in stroke cases than controls in both the non-dominant (F(2145) = 290.7, *p* < 0.001) and dominant (F(2145) = 13.8, *p* < 0.001) hands (Table 1). Six of 23 participants with AIS (median age: 10 years, 2 females) had evidence of visuospatial neglect (BIT <130) and this was associated with performance on the robotic kinesthesia task in all parameters. All six of these participants failed four or more robotic parameters in both vision conditions.

## Discussion

Children with hemiparetic CP typically demonstrate unilaterally impaired sensory and motor function in the upper extremity that can result in learned non-use of the affected limb [41]. While it is recognized that sensory dysfunction is common following stroke, accurate evaluation of sensory deficits has been limited by poor clinical diagnostic measures [14, 30, 31]. Overall, clinical assessments of proprioceptive loss following stroke lack consistency and objective tools are needed to better quantify sensory loss [42]. Advances in robotic technology provide objective, precise and reliable methods for characterizing sensory function in healthy and diseased populations [19, 27, 29, 43–46]. The KINARM robot has been used extensively in adult stroke where detailed measures of sensorimotor function have been established [7, 8, 24–26]. In this study, we identified kinesthetic deficits in children with perinatal stroke and hemiparetic CP using the aforementioned robotic exoskeleton.

Here we found that kinesthetic deficits are more common and present with greater severity in children with arterial rather than venous lesions. These results further support the idea that lesion type and location are an important determinant of proprioceptive deficits in children with perinatal stroke [16]. Overall, children with perinatal stroke moved more slowly, with greater angular deviations, difficulty when matching the speed of the robot, and difficulty matching the length of movements made by the robot. These results have many functional implications when focusing rehabilitation following stroke. While most strategies have focused on improving motor function, if a participant has difficulty sensing the direction and motion of their body, any motor gains may have limited impact to an individual’s overall function.

Using the Kinesthetic Sensitivity Test, studies have shown robust and detectable development of kinesthesia despite the relative unreliablilty of this measure [47]. In typically developing children and adolescents, studies have shown that kinesthetic acuity improves with increasing age [47]. These findings reflect those found in the present study, where the affects of age on the performance of all eight kinesthetic parameters have been illustrated. Our use of both typically developing controls and children with CP secondary to perinatal stroke, coupled with a more objective measure of kinesthetic function, gave us the unique opportunity to examine the developmental trends of kinesthesia. In general, the older a healthy control participant, the lower their response latency and angular deviation, and the better they matched the speed and length of movements made by the passive, robot-moved arm. These findings reflect improvement of kinesthetic function with age. Similarly to controls, we found that participants with CP followed relatively the same patterns of improving performance with age, however, several cases fell outside the normative ranges in each kinesthetic parameter. This indicates that although kinesthetic acuity improves with age, early brain injuries such as a perinatal stroke can cause impairments in limb motion sense.

Recent studies have quantified kinesthetic impairments in adult stroke and have shown significant correlations of robotic parameters with clinical measures of sensorimotor function and functional ability [7, 8]. Similar to adults, children with perinatal stroke demonstrated significant difficulty not only in matching the speed of the robot, but also in consistently initiating movements. Interestingly, data from healthy controls indicated that children and adolescents initiate movement faster (i.e. shorter response latency) than healthy adults examined in a different study [7] (mean RL children: 0.36 s; median RL adults: 0.40 s) when responding to the movement of the robot. The implications of these findings are not known, but could be due to lower arm inertia in the pediatric population, or lack of inhibition of movement as the frontal lobes may not have fully matured [48, 49].

The current study had subjects perform the kinesthesia task with and without vision with the hypothesis that vision could improve task performance. The idea that vision improves proprioception is a fundamental idea in rehabilitation teaching [50] since vision is thought to provide an external frame of reference for the execution of skilled movement [51, 52]. However, the AIS and PVI groups had little improvement on the kinesthesia task with vision. In fact, some subjects performed worse when vision was restored, contrary to long-standing beliefs that vision compensates for proprioception in the absence of sensory feedback. While some stroke cases corrected their performance with vision, these corrections did not bring the performance of the AIS and PVI groups to normal range. Perinatal stroke can damage cortical and subcortical areas essential for normal sensory awareness (i.e. sensations of pain, pressure, proprioception) resulting in many functional implications on an individual’s ability to perform coordinated, skilled and independent movements, potentially reducing quality of life [53, 54]. Future studies using neuroimaging may be helpful in better understanding the networks underlying proprioceptive and visual integration.

One challenge with the current robotic assessment is that it requires intact motor function of the ipsilesional arm. Both hemispheres provide contralateral and some ipsilateral projections to the limbs; therefore, strokes of the left or right hemisphere can result in deficits in the ipsilesional upper limbs [55]. Ipsilesional motor deficits can occur post-stroke and persist chronically, affecting quality of movement [56]. Four AIS and 2 PVI stroke cases demonstrated motor impairments in their ipsilesional arm as deteremined by the CMSA. When we removed these 6 cases from our group analyses, we found few changes to the results above, with the exception of PLRv in both vision conditions, where the AIS and PVI groups were no longer different from each other. As we still see issues with task performance in children without ipsilesional motor deficits, this would strongly suggest the problems we identify are occurring as a result of sensory issues or alternatively with the integration of sensory information to generate a motor plan. A recent lesion analysis by our group in 142 adults with stroke demonstrates that a large network of brain areas may be involved in performance of this task [57]. Our findings in that study suggest that the brain structures required to receive, integrate and act on kinesthetic information are far more diffuse than traditionally assumed. Further, primary sensory deficits and deficits with sensory motor integration may be challenging to separate in behavioural paradigms such as the one we have used in the present paper.

In hemiparetic CP, reduced and altered patterns of spontaneous movement may be due to abnormal sensory feedback, altered cortical reorganization of sensorimotor function and/or abnormal sensory-motor integration. These can all lead to asymmetric somatosensory processing deficits [58]. Several studies in healthy and stroke groups have discussed the lateralization of sensorimotor function in the hemispheres, where the left hemisphere is associated with visual feedback [52] and initial trajectory features (i.e. movement direction, peak acceleration, torque) [56, 59] while the right hemisphere is associated with proprioceptive feedback, limb position, and posture [19, 52, 56, 59, 60]. In this study, there were no differences in kinesthetic performance between participants that had left versus right hemispheric damage in the AIS or PVI groups. It is possible that greater neuroplasticity in early development and reorganization after injury may explain these findings. Animal studies have shown better outcomes in younger primates following unilateral lesions in the motor cortex [61]. Previous neuroimaging studies have reported significant alterations in white matter pathways connecting to the somatosensory cortex, suggesting that CP disrupts sensory and motor pathways [58, 62, 63]. One study of children with unilateral CP found that proprioception, pain and touch sensitivity did not differ between children with left versus right motor impairments, but lip and thumb stimulation in the left motor impaired group elicited smaller beta power and more symmetrical somatosensory evoked potential amplitude, suggesting different mechanisms of sensorimotor reorganization [58].

Over 50% of adults with acute stroke present with sensory deficits, yet sensory rehabilitation is often overlooked [8, 9, 53]. In children with hemiparetic CP, interventions have focused primarily on modified constraint-induced movement therapy (CIMT) and hand-arm bimanual intensive therapy (HABIT) to improve motor connectivity in the ipsilesional hemisphere, and improve use and range of movement of the paretic limb. While robotic technology offers the ability to accurately assess kinesthesia, it also provides the potential for treatments that are not easily administered in a traditional rehabilitation setting. Robotic technology provides the opportunity for sensory retraining, with a number of studies piloting robotic training to improve motor and sensory deficits in children and adults following stroke and other neurological conditions [46, 64, 65]. Robotic proprioceptive training aims to improve proprioceptive function through the use of somatosensory signals (tactile or proprioceptive afferents) in the absence of vision [65].

## Conclusions

These results have implications for perinatal stroke rehabilitation in children with sensory deficits. Traditional strategies focus on improving motor function and independence. While improving motor function may result in functional gains for some children, others may have impaired proprioception where therapy targeted at improving motor function may lead to only small functional gains. For those children with proprioceptive deficits, targeted rehabilitative training to specifically improve sensory function has significant potential to improve outcomes.

## Abbreviations

AHA: Assisting hand assessment
AIS: Arterial ischemic stroke
BIT: Behavioural inattention test
CIMT: Constraint-induced movement therapy
CMSA: Chedoke-McMaster stroke assessment
CP: Cerebral palsy
HABIT: Hand-arm bimanual intensive therapy
IDE: Initial direction error
IDEv: Initial direction error variability
KINARM: Kinestiological instrument for normal and altered reaching movements
MACS: Manual abilities classification system
PLR: Path length ratio
PLRv: Path length ratio variability
PPB: Purdue pegboard
PSOM: Pediatric stroke outcome measure
PSR: Peak speed ratio
PSRv: Peak speed ratio variability
PVI: Periventricular venous infarction
RL: Response latency
RLv: Response latency variability
TLT: Thumb localization task

## References

[1] Sherrington C (1907). On the proprioceptive system, especially in its reflex aspect. Brain.

[2] McCloskey DI (1978). Kinesthetic sensibility. Physiol Rev.

[3] Gandevia SC, Refshauge KM, Collins DF (2002). Proprioception: peripheral inputs and perceptual interactions. Adv Exp Med Biol.

[4] Sainburg RL, Ghilardi MF, Poizner H, Ghez C (1995). Control of limb dynamics in normal subjects and patients without proprioception. J Neurophysiol.

[5] Gordon J, Ghilardi MF, Ghez C (1995). Impairments of reaching movements in patients without proprioception. I Spatial errors. J Neurophysiol.

[6] Feys H, De WW, Nuyens G, van de WA, Selz B, Kiekens C (2000). Predicting motor recovery of the upper limb after stroke rehabilitation: value of a clinical examination. Physiother Res Int.

[7] Semrau JA, Herter TM, Scott SH, Dukelow SP (2013). Robotic identification of kinesthetic deficits after stroke. Stroke J Cereb Circ.

[8] Semrau JA, Wang JC, Herter TM, Scott SH, Dukelow SP (2015). Relationship between visuospatial neglect and kinesthetic deficits after stroke. Neurorehabil Neural Repair.

[9] Connell LA, Lincoln NB, Radford KA (2008). Somatosensory impairment after stroke: frequency of different deficits and their recovery. Clin Rehabil.

[10] Raju TN, Nelson KB, Ferriero D, Lynch JK (2007). Ischemic perinatal stroke: summary of a workshop sponsored by the National Institute of Child Health and Human Development and the National Institute of Neurological Disorders and Stroke. Pediatrics.

[11] Kirton A, deVeber G, Pontigon AM, MacGregor D, Shroff M (2008). Presumed perinatal ischemic stroke: vascular classification predicts outcomes. Ann. Neurol.

[12] Kirton A (2013). Predicting developmental plasticity after perinatal stroke. Dev Med Child Neurol.

[13] Tizard JP, Paine RS, Crothers B (1954). Disturbances of sensation in children with hemiplegia. J Am Med Assoc.

[14] Van Heest AE, Putnam M, House J (1993). Sensibility deficiencies in the hands of children with spastic hemiplegia. JHand SurgAm.

[15] Brown JV, Schumacher U, Rohlmann A, Ettlinger G, Schmidt RC, Skreczek W (1989). Aimed movements to visual targets in hemiplegic and normal children: is the “good” hand of children with infantile hemiplegia also normal?. Neuropsychologia.

[16] Kuczynski AM, Dukelow SP, Semrau JA, Kirton A (2016). Robotic quantification of position sense in children with perinatal stroke. Neurorehabil Neural Repair.

[17] Bleyenheuft Y, Gordon AM (2013). Precision grip control, sensory impairments and their interactions in children with hemiplegic cerebral palsy: a systematic review. Res Dev Disabil.

[18] Cooper J, Majnemer A, Rosenblatt B, Birnbaum R (1995). The determination of sensory deficits in children with hemiplegic cerebral palsy. J Child Neurol.

[19] Goble DJ, Hurvitz EA, Brown SH (2009). Deficits in the ability to use proprioceptive feedback in children with hemiplegic cerebral palsy. Int J Rehabil Res.

[20] Pickett K, Konczak J (2009). Measuring kinaesthetic sensitivity in typically developing children. Dev Med Child Neurol.

[21] Bairstow PJ, Laszlo JI (1981). Kinaesthetic sensitivity to passive movements and its relationship to motor development and motor control. Dev Med Child Neurol.

[22] Elliott JM, Connolly KJ, Doyle AJ (1988). Development of kinaesthetic sensitivity and motor performance in children. Dev Med Child Neurol.

[23] Lincoln N. The unreliability of sensory assessments. Clin Rehabil. 1991;5:273–82.

[24] Coderre AM, Zeid AA, Dukelow SP, Demmer MJ, Moore KD, Demers MJ (2010). Assessment of upper-limb sensorimotor function of subacute stroke patients using visually guided reaching. Neurorehabil Neural Repair.

[25] Dukelow SP, Herter TM, Moore KD, Demers MJ, Glasgow JI, Bagg SD (2010). Quantitative assessment of limb position sense following stroke. Neurorehabil Neural Repair.

[26] Dukelow SP, Herter TM, Bagg SD, Scott SH (2012). The independence of deficits in position sense and visually guided reaching following stroke. J Neuroengineering Rehabil.

[27] Hughes CML, Tommasino P, Budhota A, Campolo D (2015). Upper extremity proprioception in healthy aging and stroke populations, and the effects of therapist- and robot-based rehabilitation therapies on proprioceptive function. Front Hum Neurosci.

[28] Volpe BT, Huerta PT, Zipse JL, Rykman A, Edwards D, Dipietro L (2009). Robotic devices as therapeutic and diagnostic tools for stroke recovery. Arch Neurol.

[29] Maciejasz P, Eschweiler J, Gerlach-Hahn K, Jansen-Troy A, Leonhardt S (2014). A survey on robotic devices for upper limb rehabilitation. J Neuroengineering Rehabil.

[30] Garraway WM, Akhtar AJ, Gore SM, Prescott RJ, Smith RG (1976). Observer variation in the clinical assessment of stroke. Age Ageing.

[31] Carey LM, Oke LE, Matyas TA (1996). Impaired limb position sense after stroke: a quantitative test for clinical use. Arch Phys Med Rehabil.

[32] Kitchen L, Westmacott R, Friefeld S, MacGregor D, Curtis R, Allen A (2012). The pediatric stroke outcome measure: a validation and reliability study. Stroke.

[33] Eliasson AC, Krumlinde-sundholm L, Rosblad B, Beckung E, Arner M, Ohrvall AM (2006). The Manual Ability Classification System (MACS) for children with cerebral palsy: scale development and evidence of validity and reliability. Dev Med Child Neurol.

[34] Bohannon RW, Smith MB (1987). Interrater reliability of a modified Ashworth scale of muscle spasticity. Phys Ther.

[35] Hirayama K, Fukutake T, Kawamura M (1999). “Thumb localizing test” for detecting a lesion in the posterior column-medial lemniscal system. J Neurol Sci.

[36] Krumlinde-sundholm L, Holmefur M, Kottorp A, Eliasson AC (2007). The Assisting Hand Assessment: current evidence of validity, reliability, and responsiveness to change. Dev Med Child Neurol.

[37] Barreca SR, Stratford PW, Lambert CL, Masters LM, Streiner DL (2005). Test-retest reliability, validity, and sensitivity of the Chedoke arm and hand activity inventory: a new measure of upper-limb function for survivors of stroke. Arch Phys Med Rehabil.

[38] Tiffin J, Asher EJ (1948). The Purdue pegboard; norms and studies of reliability and validity. J Appl Psychol.

[39] Oldfield RC (1971). The assessment and analysis of handedness: the Edinburgh inventory. Neuropsychologia.

[40] Wilson B, Cockburn J, Halligan P (1998). Development of a behavioral test of visuospatial neglect. Arch Phys Med Rehabil.

[41] Boyd R, Sakzewski L, Ziviani J, Abbott DF, Badawy R, Gilmore R (2010). INCITE: a randomised trial comparing constraint induced movement therapy and bimanual training in children with congenital hemiplegia. BMC Neurol.

[42] Doyle S, Bennett S, Fasoli SE, McKenna KT. Interventions for sensory impairment in the upper limb after stroke. Cochrane Database Syst Rev. 2010;6:CD006331. 10.1002/14651858.CD006331.pub2 PMC6464855 20556766

[43] Goble DJ, Aaron MB, Warschausky S, Kaufman JN, Hurvitz EA (2012). The influence of spatial working memory on ipsilateral remembered proprioceptive matching in adults with cerebral palsy. Exp Brain Res.

[44] Debert CT, Herter TM, Scott SH, Dukelow S (2012). Robotic assessment of sensorimotor deficits after traumatic brain injury. J Neurol Phys Ther JNPT.

[45] Scott SH, Dukelow SP (2011). Potential of robots as next-generation technology for clinical assessment of neurological disorders and upper-limb therapy. J Rehabil Res Dev.

[46] Fasoli SE, Ladenheim B, Mast J, Krebs HI (2012). New horizons for robot-assisted therapy in pediatrics. Am J Phys Med Rehabil Assoc Acad Physiatr.

[47] Visser J, Geuze RH (2000). Kinaesthetic acuity in adolescent boys: a longitudinal study. Dev Med Child Neurol.

[48] Tamnes CK, Ostby Y, Fjell AM, Westlye LT, Due-Tønnessen P, Walhovd KB (2010). Brain maturation in adolescence and young adulthood: regional age-related changes in cortical thickness and white matter volume and microstructure. Cereb Cortex N Y N 1991.

[49] Lebel C, Walker L, Leemans A, Phillips L, Beaulieu C (2008). Microstructural maturation of the human brain from childhood to adulthood. Neuroimage.

[50] Smorenburg ARP, Ledebt A, Deconinck FJA, Savelsbergh GJP (2011). Visual feedback of the non-moving limb improves active joint-position sense of the impaired limb in Spastic Hemiparetic Cerebral Palsy. Res Dev Disabil.

[51] Goodale MA, Westwood DA, Milner AD (2004). Two distinct modes of control for object-directed action. Prog Brain Res.

[52] Goble DJ, Brown SH (2008). The biological and behavioral basis of upper limb asymmetries in sensorimotor performance. Neurosci Biobehav Rev.

[53] Schabrun SM, Hillier S (2009). Evidence for the retraining of sensation after stroke: a systematic review. Clin Rehabil.

[54] Scott SH (2012). The computational and neural basis of voluntary motor control and planning. Trends Cogn Sci.

[55] Schaefer SY, Haaland KY, Sainburg RL (2009). Hemispheric specialization and functional impact of ipsilesional deficits in movement coordination and accuracy. Neuropsychologia.

[56] Schaefer SY, Haaland KY, Sainburg RL (2007). Ipsilesional motor deficits following stroke reflect hemispheric specializations for movement control. Brain J Neurol.

[57] Kenzie JM, Semrau JA, Findlater SE, Yu AY, Desai JA, Herter TM (2016). Localization of Impaired Kinesthetic Processing Post-stroke. Front Hum Neurosci.

[58] Riquelme I, Padrón I, Cifre I, González-Roldán AM, Montoya P (2014). Differences in somatosensory processing due to dominant hemispheric motor impairment in cerebral palsy. BMC Neurosci.

[59] Sainburg RL, Schaefer SY (2004). Interlimb differences in control of movement extent. J Neurophysiol.

[60] Goble DJ, Brown SH (2007). Task-dependent asymmetries in the utilization of proprioceptive feedback for goal-directed movement. Exp Brain Res.

[61] Kennard MA (1936). Age and other factors in motor recovery from precentral lesions in monkeys. Am J Physiol.

[62] Hoon AH, Stashinko EE, Nagae LM, Lin DDM, Keller J, Bastian A (2009). Sensory and motor deficits in children with cerebral palsy born preterm correlate with diffusion tensor imaging abnormalities in thalamocortical pathways. Dev Med Child Neurol.

[63] Hoon AH, Lawrie WT, Melhem ER, Reinhardt EM, Van Zijl PCM, Solaiyappan M (2002). Diffusion tensor imaging of periventricular leukomalacia shows affected sensory cortex white matter pathways. Neurology.

[64] Cappello L, Elangovan N, Contu S, Khosravani S, Konczak J, Masia L (2015). Robot-aided assessment of wrist proprioception. Front Hum Neurosci.

[65] Aman JE, Elangovan N, Yeh I-L, Konczak J (2014). The effectiveness of proprioceptive training for improving motor function: a systematic review. Front Hum Neurosci.

